# Population Census of a Large Common Tern Colony with a Small Unmanned Aircraft

**DOI:** 10.1371/journal.pone.0122588

**Published:** 2015-04-15

**Authors:** Dominique Chabot, Shawn R. Craik, David M. Bird

**Affiliations:** 1 Department of Natural Resource Sciences, McGill University, Ste-Anne-de-Bellevue, Québec, Canada; 2 Département des Sciences, Université Sainte-Anne, Pointe-de-l’Église, Nova Scotia, Canada; 3 Avian Science and Conservation Centre of McGill University, Ste-Anne-de-Bellevue, Québec, Canada; Phillip Island Nature Parks, AUSTRALIA

## Abstract

Small unmanned aircraft systems (UAS) may be useful for conducting high-precision, low-disturbance waterbird surveys, but limited data exist on their effectiveness. We evaluated the capacity of a small UAS to census a large (>6,000 nests) coastal Common tern (*Sterna hirundo*) colony of which ground surveys are particularly disruptive and time-consuming. We compared aerial photographic tern counts to ground nest counts in 45 plots (5-m radius) throughout the colony at three intervals over a nine-day period in order to identify sources of variation and establish a coefficient to estimate nest numbers from UAS surveys. We also compared a full colony ground count to full counts from two UAS surveys conducted the following day. Finally, we compared colony disturbance levels over the course of UAS flights to matched control periods. Linear regressions between aerial and ground counts in plots had very strong correlations in all three comparison periods (*R* ^2^ = 0.972–0.989, *P* < 0.001) and regression coefficients ranged from 0.928–0.977 terns/nest. Full colony aerial counts were 93.6% and 94.0%, respectively, of the ground count. Varying visibility of terns with ground cover, weather conditions and image quality, and changing nest attendance rates throughout incubation were likely sources of variation in aerial detection rates. Optimally timed UAS surveys of Common tern colonies following our method should yield population estimates in the 93–96% range of ground counts. Although the terns were initially disturbed by the UAS flying overhead, they rapidly habituated to it. Overall, we found no evidence of sustained disturbance to the colony by the UAS. We encourage colonial waterbird researchers and managers to consider taking advantage of this burgeoning technology.

## Introduction

Great effort and resources are invested worldwide in monitoring waterbird populations, which have long had important symbolic and functional value, historically in the context of recreational hunting and more recently as indicators of ecosystem health [1–4]. Monitoring relies heavily on aerial surveys, which are convenient in aquatic environments and can rapidly cover large areas [5,6]. However, ground surveys are also routinely carried out for smaller or more cryptic species, or to gather more precise data over relatively small areas. In addition to the time and effort required in the field, investigator disturbance is a well-documented drawback of ground-based monitoring [7–11], although colonial waterbirds can habituate to some degree to regular research disturbance [12].

In recent years, there has been increasing interest in the use of small unmanned aircraft systems (UAS) for surveying birds [13–17]. The perceived potential of UAS is that they can approach the precision of ground surveys thanks to their low-altitude, high-resolution (<10 cm/pixel) aerial imaging capabilities while avoiding the disturbance associated with ground surveys thanks to their small size (<3-m wingspan) and quiet electric motors. Moreover, they can be deployed in a timely fashion over small (<3-km radius) aquatic environments that might otherwise be challenging to access or navigate at ground level. Whereas conventional aerial surveys of waterbirds have been the subject of numerous accuracy studies [18–22], only two studies to date have presented quantitative survey data for entire flocks or colonies obtained using UAS and compared them to ground counts (staging flocks of geese [16]; breeding colony of gulls [17]), and in both cases small sample sizes precluded statistical analyses. Therefore, there is a need to more rigorously assess the ability of UAS to achieve their perceived potential in order for the broader research and management community to appreciate the performance of this emerging technology.

In 2012, we evaluated a small “off-the-shelf” UAS for the purpose of conducting a population census of the third largest Common tern (*Sterna hirundo*) breeding colony in North America (up to ~7,400 nests), situated on a coastal barrier island complex in Kouchibouguac National Park (KNP), New Brunswick, Canada. This site represents a prime example of where UAS surveys could be beneficial, as the annual ground-based census conducted by the park causes sustained disturbance to the colony for several hours during peak incubation as terns flush off their nests and relentlessly mob surveyors. In addition, surveyors inadvertently trample nests, walk all over the fragile beach grass habitat and disturb a population of Red-breasted mergansers (*Mergus serrator*) counting upwards of 80 nests throughout the tern colony, which are known to occasionally abandon their nests following investigator disturbance [23]. Our study objectives were to (1) compare UAS aerial counts to ground counts over a multi-day period, (2) assess sources of variation and error in aerial/ground detection rates, (3) determine a reliable coefficient allowing estimation of the number of nests in the colony based on the number of terns detected in the aerial imagery, and (4) assess how much disturbance, if any, the UAS causes to the colony.

## Materials and Methods

### Study area

**Fig 1:**
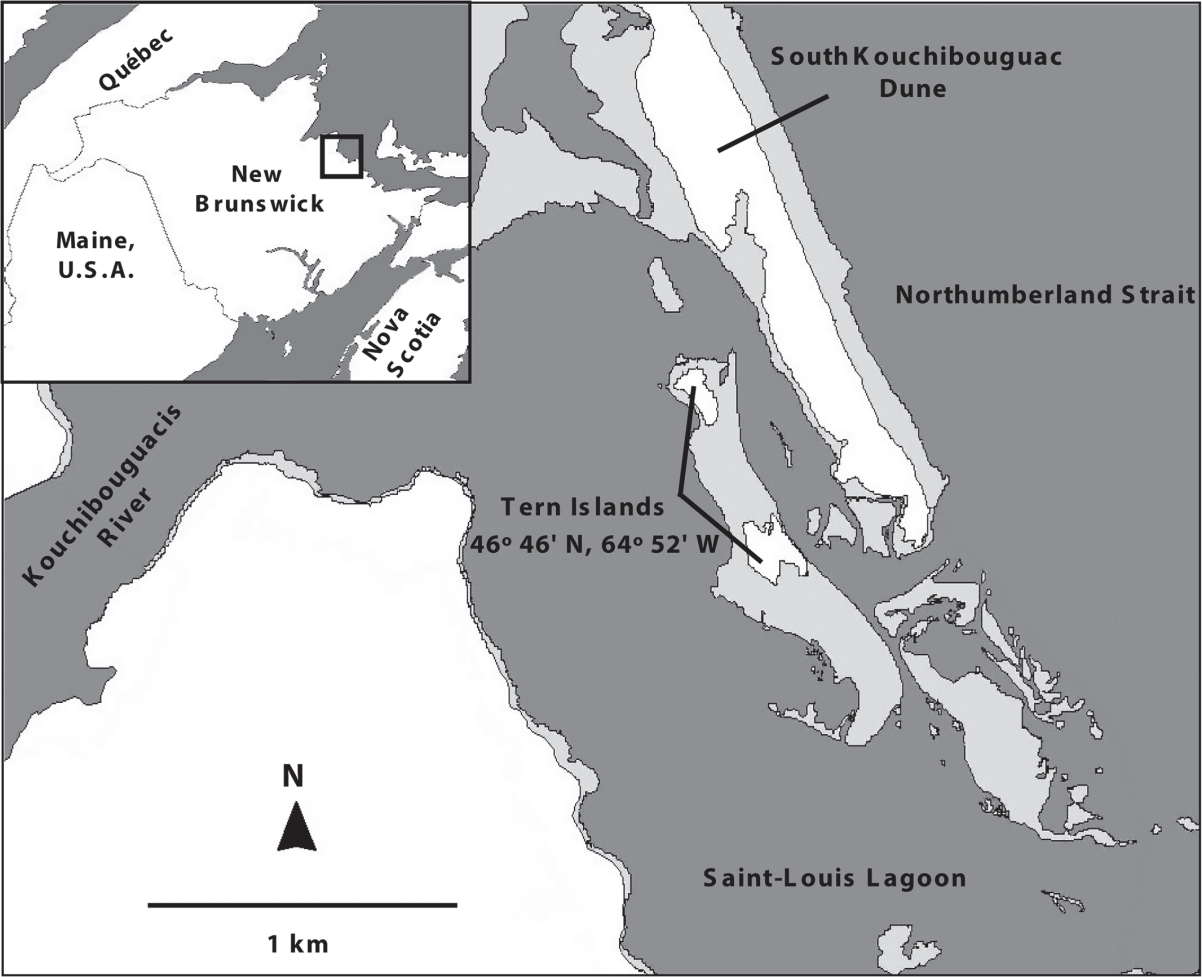
Map of the Tern Islands study area in Kouchibouguac National Park, New Brunswick. Light gray areas represent intertidal zones (figure adapted from Craik and Titman [23]).

**Fig 2:**
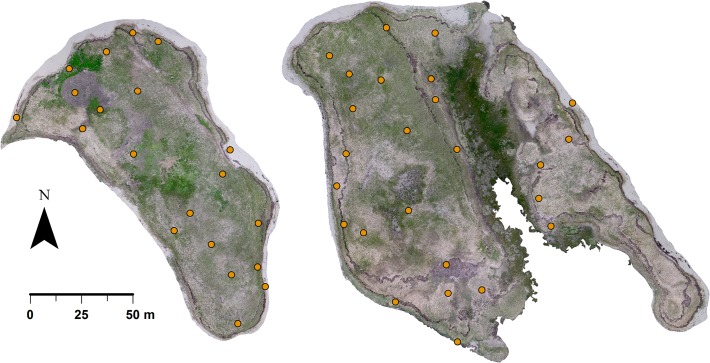
Tern Islands (left: Island 1; right: Island 2), Kouchibouguac National Park, New Brunswick, 2012. Imagery was constructed from seamless mosaicking of overlapping aerial photos with a ground resolution of ~3 cm/pixel acquired from 91 m in altitude by a small unmanned aircraft system (UAS). Light and dull green areas contain grass; darker and more vivid green areas contain forbs; beige and light gray areas contain dead grass; darker gray-brown areas are mostly comprised of washed-up Eelgrass and some dead forbs; and very light sandy beach areas are visible around the periphery of the islands. Orange dots indicate locations of 5-m-radius plots in which aerial Common tern counts from the UAS imagery were compared to nest counts conducted on the ground.

The KNP Common tern colony is located on Tern Islands, a dynamic barrier sand island complex in the Saint-Louis Lagoon currently composed of two main islands (hereby referred to as Island 1, to the north, and Island 2, to the south) ~300 m apart, ~800 m off the east coast of New Brunswick, totaling ~3 ha in size (Fig 1). Topography on the islands is low-lying (mostly <2 m ASL) and sand is mostly stabilized by Marram grass (*Ammophila breviligulata*) and lesser amounts of Sea lyme grass (*Leymus mollis*) in lower-lying areas, as well as scattered Common yarrow (*Achillea millefolium*), Pigweed (*Chenopodium album*) and Tumble mustard (*Sisymbrium altissimum*). Vast mats of dead vegetation covered parts of the islands in 2012 and strips of Eelgrass (*Zostera marina*) deposited by very high tides and storm surges were strewn parallel to the shore around the outer areas of the islands (Fig 2). As the largest Common tern colony in Canada, Parks Canada has closely monitored its population over the past four decades by means of annual total nest counts performed on the ground. The terns generally nest in relatively open areas (e.g. on mats of dead vegetation or Eelgrass) as opposed to densely vegetated areas of the islands.

### Ground surveys

At the height of the 2012 incubation period when nest numbers peak in the colony, we established 45 circular “comparison plots” of 5-m radius (20 on Island 1 and 25 on Island 2) by generating random points a minimum of 10 m apart in preexisting polygons of Tern Islands in ArcMap v.10.1 (Esri, Redlands, CA, USA). Collectively, the plots sampled a variety of habitats present on the islands (Fig 2). We used a handheld Garmin GPS to locate the points on site and marked them with ~1-m wooden stakes. We placed an orange or yellow plastic cone atop each stake and subsequently recorded their locations using a high-accuracy Trimble Pathfinder backpack GPS unit (10-60-cm positional error reported throughout data collection).

We conducted total counts of active nests in each plot on 17, 21 and 25 June by extending a non-stretching string from the stake and positioning two surveyors at the 2.5-m and 5-m marks, respectively. The surveyors walked in a circle around the stake while keeping the string under tension, the former surveyor counting all nests between himself and the stake and the latter counting nests between himself and the first surveyor. Nests were counted if they contained at least one egg or at least one chick in or immediately next to them. On 21 June, Parks Canada conducted a total nest count throughout the colony as part of its ongoing population monitoring program, which involved a dozen surveyors positioned side-by-side 2 m apart along a rope walking back-and-forth transects over the entire area of Tern Islands [24].

### Aerial surveys

**Fig 3:**
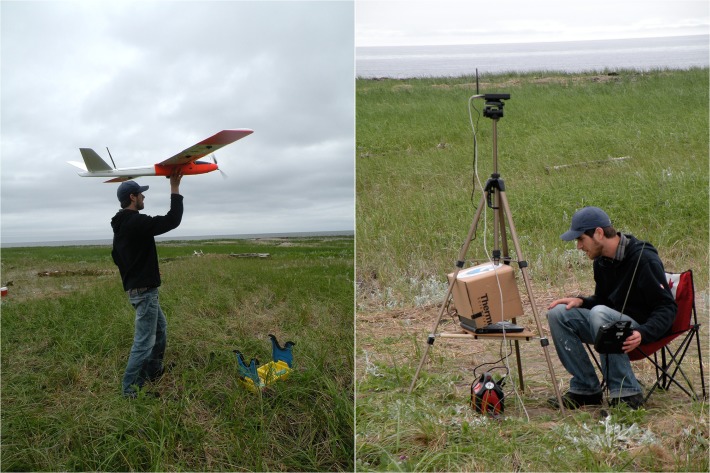
The AI-Multi electric unmanned aircraft and ground control station. Shown on the left prior to hand-launch from the South Kouchibouguac Dune, Kouchibouguac National Park, New Brunswick, 2012. The ground control station is composed of a laptop and radio modem mounted on a modified camera tripod, as well as a portable power supply.

We conducted aerial surveys of Tern Islands using the AI-Multi UAS (by Aerial Insight, Brandon, MB, Canada), a 2.1-m wingspan, 4-kg hand-launched electric airplane (Fig 3, S1 Video) equipped with a gimbaled downward-pointing camera and a miniature autopilot system (MP2028g by MicroPilot, Stony Mountain, MB, Canada) that executes pre-programmed flights created with the MicroPilot Horizon software package. The aircraft was powered by an 11,000 mAh rechargeable lithium-polymer battery, which can provide up to 45–60 min of flight endurance at an airspeed of ~60 km/h depending on wind conditions. It can withstand wind speeds of ≤30 km/h but it cannot operate in precipitation. During flight, the aircraft is tracked via radio link from a laptop-based ground control station (Fig 3) on which Horizon displays a moving map and various flight and system parameters through a “virtual cockpit” interface. New commands can be transmitted to the aircraft in mid-flight (e.g. course adjustments, abort mission) and it is also possible to override the autopilot and control the aircraft manually using a standard radio-control transmitter. Following course completion, the aircraft performs an autonomous belly-landing at a user-specified location (S1 Video), which requires a vegetated surface and a 50–100 m (depending on wind conditions) runway clear of obstacles. The modular aircraft and field kit can be transported in an economy-size car. The system, including accessories (e.g. camera, radio transmitter, laptop, portable power supply, spare batteries and chargers, field repair kit), costs ~US$20,000, and with proper use and maintenance can complete >200 flights before eventually requiring full airframe replacement (~$2,000), mainly due to the accumulated wear sustained on landings.

From 16–25 June, weather permitting, we performed a total of 12 flights (two on 16 June, one on 17 June, one on 18 June, three on 19 June, two on 22 June, and three on 25 June) lasting 25 min on average, all between 07:00 and 16:30. We transported the components of the UAS by motorboat to the South Kouchibouguac Dune directly across from Tern Islands (Fig 1) and assembled the system on site. For each flight, the aircraft was programmed to fly repeated back-and-forth (northward-southward) transects over the islands, making six passes at an altitude of 91 m (300 ft) followed by six more at 122 m (400 ft) before returning to land on the dune. While the aircraft was over the islands, the autopilot-triggered camera (10-megapixel Canon PowerShot S90) shot a continuous series of overlapping photos with the lens fully zoomed out (28mm focal length), the focus set to infinity, the shutter speed set to 1/1,250 sec, and the aperture and ISO on an automatic setting that favored opening the aperture as much as possible (*f*/2.0) before increasing the ISO sensitivity beyond the minimum value of 80. The spatial resolution of photos shot from 91 m in altitude was ~3 cm/pixel (S1 Photo) and those from 122 m was ~4 cm/pixel.

We examined the aerial photos on computer and selected the clearest 91-m shots from each flight on 17, 22 and 25 June to construct photomosaics of the entire islands. Coverage of Island 2 was incomplete in the 91-m imagery from 17 and 22 June, so we filled in the gaps with 122-m imagery. The photos were stitched together using PTGui v.9.1 (New House Internet Services BV, Rotterdam, Netherlands) by first manually identifying a series of matching points in pairs of photos and then allowing the program to automatically perform fine-scale alignment, rectification and seamless blending of the images. In this manner, mosaics of Island 1 were created from four to five individual photos and mosaics of Island 2 were created from seven to nine photos (Fig 2). We then loaded the images into ArcMap and georeferenced them by linking the plastic cones placed atop the 45 stakes marking the comparison plots to their respective coordinates recorded by the Trimble GPS unit. Upon applying second-order polynomial transformations to the images to improve the alignment of the cones with their specified coordinates, the root-mean-square error (average distance between the center of cones and the recorded coordinates) ranged from 32–37 cm on Island 1 and 29–55 cm on Island 2.

**Fig 4:**
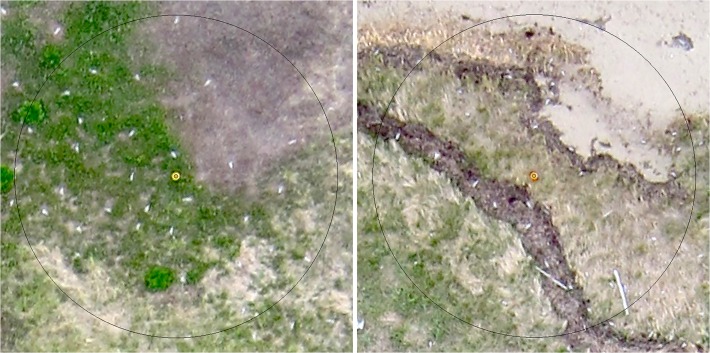
Example images of 5-m-radius plots acquired by a small unmanned aircraft system (UAS). Plots were used to compare aerial Common tern counts from the UAS imagery to nest counts conducted on the ground on Tern Islands, Kouchibouguac National Park, New Brunswick, 2012. Thirty terns were counted in the plot to the left and 36 in the plot to the right.

We extracted 5-m-radius circles centered on each cone (Fig 4) from the aerial imagery in order to make comparisons with nest counts performed on the ground, retaining the clearest shot of each comparison plot among all flights performed on each day. For consistency, we excluded five plots from Island 2 on 17 June and two plots on 22 June that were not covered by 91-m imagery. Using the count tool in Photoshop v.12.0 (Adobe, San Jose, CA, USA), we then counted the total number of terns visible in each plot in each of the three comparison periods (17 June, 21–22 June, and 25 June), with the underlying assumption that there should be at least one tern attending each nest almost all of the time [25]. In a concurrent study of habitat relationships among 105 randomly selected nests in the Tern Islands colony, the minimum observed distance to the nearest neighboring nest was 48 cm (mean = 128 cm; SD = 74 cm). Terns that appeared closer together than this distance in the aerial imagery were considered to be pairs attending a single nest and therefore only counted as one. In most cases these assumed pairs were less than a body length apart. We also excluded flying terns from counts. We then used the same approach to carry out total tern counts over the entire colony in the imagery from each of the two flights performed on 22 June (the day after the total nest count conducted by Parks Canada), in this case also counting terns in the portions filled in by 122-m imagery. We did not count terns located <1 m from the wrack line representing the high tide line as none were seen nesting this close to the water in the field and it was common for terns to loaf near the outer edge of the beach and in the shallow waters around the periphery of the islands.

When counting terns in the aerial imagery, we suspected that ground cover influenced their detectability. In particular, they contrasted more poorly with very pale dead vegetation (primarily dead grass). To investigate this, we conducted supervised classification of ground cover using ArcMap’s Spatial Analyst extension (method developed, validated and described in detail by Chabot and Bird [26]) in the imagery from 25 June (which was deemed to be of the highest quality) by first manually drawing a series of polygons serving as “training samples” to create spectral signatures for various cover classes (using photos taken on the ground to guide identification), then allowing the program to automatically classify the entire area of the islands into a final set of five classes: sand, grass, forbs, pale dead vegetation, and dark dead vegetation (mostly Eelgrass). We then used FRAGSTATS [27] to calculate percent cover by class in each comparison plot.

### Disturbance observations

In order to assess possible disturbance to the colony by the aircraft, we recorded disturbance levels on each island during 10 flights (we did not collect data during the first two flights on 16 June as these were considered practice runs) as well as 10 matched control periods of equal duration starting 10 minutes after each flight. An observer located on the South Kouchibouguac Dune (Fig 1) with a clear view of both islands scored the overall disturbance level on each island at 30-second intervals from the moment the aircraft was launched until the moment it touched down, then did the same during control periods. Disturbance was scored as either “0” for no noticeable disturbance, “1” for moderate disturbance (localized flushing or visible agitation of terns affecting less than half of the island), or “2” for high disturbance (flushing of terns throughout most or all of the island, i.e. “upflights”, or “panics/dreads” whereby terns suddenly fall silent, rapidly fly away from the colony, then rise and start calling; [25,28]). In this manner, a total of 502 disturbance samples were recorded for each island throughout all flights and another 502 each throughout control periods (range = 47–55 samples/island/flight and control period). For each flight and control period, we summed the disturbance scores of all samples across both islands in order to obtain a single total colony disturbance score.

### Statistical analyses

We performed statistical analyses with SPSS Statistics v.21.0 (IBM, Armonk, NY, USA), using a significance level of 0.05. For all analyses, we first ran Kolmogorov-Smirnov tests to confirm that variables were normally distributed. For comparison plots, we ran a linear regression analysis in each comparison period with ground counts as the independent variable and corresponding aerial counts as the dependent variable [22], and the intercept set to zero. We noted the regression coefficient (β) as well as its 95% confidence interval (95% CI), the significance of the regression (*F*, *P*), and the model fit as expressed by the coefficient of determination (*R* ^2^). To test whether ground cover, specifically pale dead vegetation, influenced tern detectability, we ran a Pearson’s correlation test in each comparison period between percent pale dead vegetation cover and the ratio of terns counted in the aerial imagery to nests counted on the ground (i.e. aerial count divided by ground count) for all plots containing ≥10 nests. Finally, we used a paired-samples *t*-test to determine if colony disturbance levels were overall different between flights and control periods.

### Ethics statement

UAS flights were authorized by Transport Canada in accordance with a Special Flight Operation Certificate (#12007), and data collection was authorized by a Parks Canada Agency Research and Collection Permit (#KOU-2012-10913) as well as a McGill University Animal Use Protocol (#5394). Kouchibouguac National Park is Canadian federal government property and the Parks Canada permit authorized us to sample Tern Islands, which is designated as sensitive habitat and off limits to the public. The individual pictured in this manuscript is the principal author and has given written informed consent (as outlined in PLOS consent form) to publish these case details.

## Results

**Fig 5:**
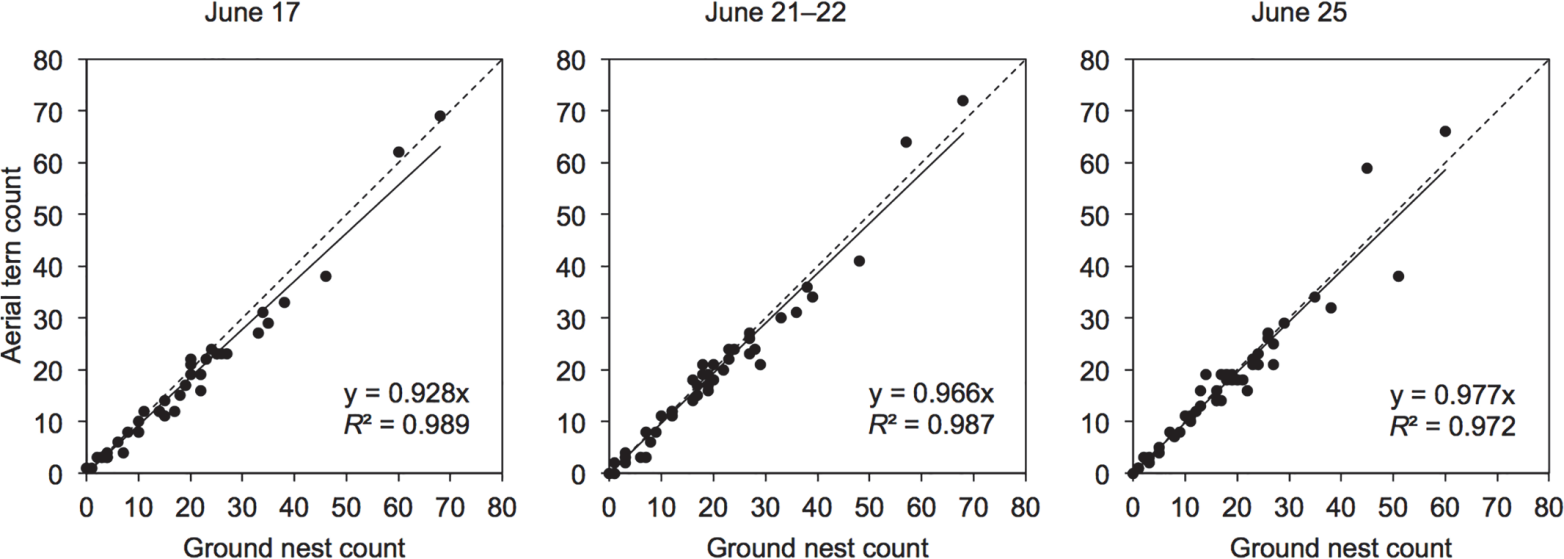
Linear regressions between aerial Common tern counts from an unmanned aircraft and ground nest counts. Counts were performed in 5-m-radius plots during three comparison periods (17 June: *n* = 40; 21–22 June: *n* = 43; 25 June: *n* = 45) on Tern Islands, Kouchibouguac National Park, New Brunswick, 2012.

Results of aerial/ground comparative surveys are summarized in Table 1. Ground nest counts in comparison plots on 17 June recorded a total of 772 nests (range = 0–68 nests/plot) among 40 plots, compared to 703 terns (91.1% of ground count; range = 1–69 terns/plot) counted in the same plots in the aerial imagery. On 21 June, 845 total nests (range = 0–68 nests/plot) were counted on the ground among 43 plots, compared to 802 terns (94.9% of ground count; range = 0–72 terns/plot) counted in the aerial imagery from 22 June. On 25 June, 806 nests (range = 0–60 nests/plot) were counted on the ground among 45 plots, compared to 782 terns (97.0% of ground count; range = 0–66 terns/plot) counted in the aerial imagery. Linear regressions between the two count methods were very strong and showed similar coefficients in all three comparison periods (Fig 5), although the coefficient increased slightly in each successive period: β_17 June_ = 0.928 (95% CI = 0.897–0.959, *F*_1,39_ = 3,647.329, *P* < 0.001, *R* ^2^ = 0.989); β_21–22 June_ = 0.966 (95% CI = 0.931–1.001, *F*_1,42_ = 3,107.002, *P* < 0.001, *R* ^2^ = 0.987); β_25 June_ = 0.977 (95% CI = 0.927–1.027, *F*_1,44_ = 1,555.357, *P* < 0.001, *R* ^2^ = 0.972).

**Table 1:** Comparison of total Common tern nest counts performed on the ground to tern counts performed in aerial imagery collected by a small unmanned aircraft system (UAS) in 5-m-radius plots as well as throughout the entire breeding colony on Tern Islands, Kouchibouguac National Park, New Brunswick, 2012.

Survey	Total ground count	Total aerial count	Aerial/ ground ratio
*Comparison plots*			
17 June (*n* = 40)	772	703	91.1%
21–22 June (*n* = 43)	845	802	94.9%
25 June (*n* = 45)	806	782	97.0%
*Full colony census* *			
21–22 June	6,329	5,924	93.6%
		5,952	94.0%

*Two full colony counts were performed in aerial imagery from back-to-back UAS flights on 22 June.

The full colony census conducted by Parks Canada on 21 June recorded 2,695 nests on Island 1 and 3,634 nests on Island 2, for a total of 6,329 nests in the colony (Table 1). In contrast, we counted 2,539 terns on Island 1 (94.2% of ground count) and 3,385 terns on Island 2 (93.1% of ground count), for a total of 5,924 terns (93.6% of ground count), in the aerial imagery from the first flight carried out on 22 June. In the imagery from the second flight, we counted 2,573 terns on Island 1 (95.5% of ground count) and 3,379 terns on Island 2 (93.0% of ground count), for a total of 5,952 terns (94.0% of ground counts).

There was no significant correlation between percent pale dead vegetation cover in comparison plots and the ratio of terns counted in aerial imagery to nests counted on the ground in any of the comparison periods (17 June: *n* = 29, *r* = 0.035, *P* = 0.855; 21–22 June: *n* = 31, *r* = -0.277, *P* = 0.132; 25 June: *n* = 33, *r* = -0.323, *P* = 0.066), although correlations were negative as expected on 21–22 June and 25 June, and came close to statistical significance in the latter period.

Total disturbance scores across both islands averaged 4.7 (SEM = 1.3, range = 0–15) per flight over the course of the 10 flights during which disturbance levels were recorded, compared to 4.8 (SEM = 2.6, range = 0–26) per matched control period. This difference was not statistically significant (*t*_9_ = -0.031, *P* = 0.976). No disturbance at all was recorded on either island during four control periods, but only during one flight. Throughout all flights we observed a total of eight upflights or panics, compared to four throughout control periods. Finally, we observed upflights shortly after the aircraft took off and first approached the colony on the first flight of the first two survey days (including 16 June when we did not record detailed disturbance data), after which this did not reoccur.

## Discussion

In this study, the AI-Multi unmanned aircraft system (UAS) proved to be an effective alternative to ground surveys for censusing the Tern Islands Common tern colony of Kouchibouguac National Park, yielding high-resolution aerial imagery in which tern counts ranged from 91–98% of nest counts performed on the ground. There are several potential explanations for the discrepancies between aerial and ground counts that are not necessarily mutually exclusive.

First, it is possible that not all terns were detected in the aerial imagery. When performing counts, we noticed that the contrast of terns was poorer over certain backgrounds (chiefly pale dead vegetation), that they were more challenging to discern in blurrier portions of the imagery, and that their visibility appeared to be affected by lighting conditions. The 17 June imagery differed from the 22 and 25 June imagery as a result of bright, sunny sky conditions during the former day compared to overcast conditions during the latter days. This yielded very clear shots but with pronounced shadows and occasional exceedingly bright, “blown-out” areas on 17 June compared to deeper, more contrasting colors and no shadows on 22 and 25 June, which we found to make terns overall more visible on the latter days. We suspect this contributed to the distinctly lower regression coefficient with ground counts on 17 June (Fig 5). We also tested for a relationship of greater proportional underestimation of numbers in aerial imagery compared to ground counts with increasing pale dead vegetation cover in comparison plots, finding no correlation on 17 June and weak, statistically insignificant correlations on 21–22 and 25 June. We believe that the results for the latter days nevertheless suggest the existence of the presumed relationship, while the lack of any correlation on 17 June may be due to the effect being masked by the overall poorer quality of the imagery as a result of lighting conditions.

Second, it is possible that not all nests counted on the ground were active. In a concurrent study of nesting success in the colony for which we monitored 105 nests for up to 3.5 weeks, we observed several instances where eggs remained in seemingly abandoned nests for multiple days, and occasionally weeks. Because terns are flushed off nests during ground counts, it would have been impossible to differentiate such nests from active ones. Therefore, while ground counts could be said to have recorded the total number of nesting *attempts*, UAS aerial surveys, because they were constrained to counting terns, could only record the number of active nests at any given time. Third, it is also possible that active nests were sometimes left unattended. Nest attentiveness is known to increase throughout egg-laying, from as little as 40% after the first egg is laid to ≥95% within a few days of clutch completion [28–30]. Among the 105 nests we monitored starting on 15 June, 11 continued laying through subsequent checks on 18 and 21 June. Reduced attendance of incomplete clutches may therefore also have contributed to the lower regression coefficient on 17 June.

The regression coefficient increase from 21–22 June to 25 June was not as large (Fig 5), but it is worth noting the substantial decrease in total nests counted on the ground, from 885 among all 45 plots on 21 June (including the two plots excluded from the comparative analysis for that period) to 806 on 25 June, whereas numbers were virtually constant from 17 to 21 June. At least part of this decrease likely resulted from underestimation, as by 25 June a significant proportion of nests had fully hatched and spotting chicks in and around nests proved more challenging than spotting eggs. In addition, surveyors were more distracted trying to avoid stepping on wandering chicks, which were often concealed in vegetation. These circumstances were especially problematic in more populous plots. Even during the first two comparison periods, nests in the two most populous plots (~60–70 nests each) were more challenging to keep a tally of using our circular survey method in which the outer surveyor scanned a total area three times as large as the inner surveyor, and we believe this is why greater numbers were counted in these plots in the aerial imagery (Fig 5). Other instances where aerial counts of plots exceeded ground counts (on 10 occasions by one, twice by two, and once by three) in the first two comparison periods may have been due to loafing terns or false positives.

Full island and colony counts yielded very similar aerial/ground ratios to comparison plot counts, especially when considering the ratios for total numbers counted among all plots: 94.9% among 43 plots in the 21–22 June period compared to 93.6% (94.2% on Island 1 and 93.1% on Island 2) throughout the entire colony in the imagery from the first flight on 22 June and 94.0% (95.5% on Island 1 and 93.0% on Island 2) in the second flight (Table 1). There could be a few reasons for the slightly lower ratios in full colony counts. First, for comparison plots we selected the clearest image of each plot between the two flights on 22 June in order to achieve the most reliable counts possible, whereas the full colony counts were performed in the imagery of each flight separately. Second, counts in each plot focused the observer on a relatively small area that was meticulously scanned, whereas equally thorough scanning of the entire area of the islands was possibly not achieved. Third, lower aerial/ground ratios in the full counts of Island 2 compared to Island 1 suggest that underestimation of tern numbers may have occurred in the portions of Island 2 filled in by lower resolution 122-m altitude imagery. A total of 425 (12.6% of island count) and 383 (11.3% of island count) terns were counted in these portions of imagery from the first and second flight, respectively.

Overall, our findings highlight how survey timing, subject visibility and weather conditions are important factors to consider for surveying colonial waterbirds with a small UAS. For Common terns, surveys should be carried out as close as possible to the peak of the incubation period, when the proportion of completed clutches is highest but before nests begin hatching en masse, in order to capture the period of highest nest attendance by adults. Following our method and provided ideal overcast sky conditions, optimally timed UAS surveys should yield Common tern population counts in the 93–96% range of the numbers of nests that would be counted on the ground. Under sunny conditions, the ratio may drop by a few percentage points. It should be noted however that a superior camera may improve overall detectability of subjects. For example, Watts et al. [15] describe a custom-built UAS capable of carrying a heavier, higher-performance single-lens reflex (SLR) camera than the compact model we used, and current SLRs now boast resolutions of up to 40 megapixels (i.e. four times the resolution of our camera).

Regarding disturbance, we found no statistically significant evidence that the UAS caused more overall disturbance to the colony over the course of 10 flights than during matched control periods when the aircraft was not airborne, although anecdotal evidence suggests that the UAS likely caused some limited disturbance. It is generally difficult to objectively quantify disturbance levels across a large tern colony, and we opted for a simple scoring scheme at fixed time intervals because we felt that added complexity might end up compounding subjective assumptions. Disturbance and agitation in Common tern colonies are common even in the absence of investigators; causes include fighting among neighboring individuals as well as the presence of terrestrial or avian predators. Although upflights and panics are often caused by the latter, they also regularly occur for no discernible reason [25,28,31]. We observed a total of eight upflights or panics throughout flights compared to four during control periods, which may suggest the aircraft was occasionally the cause. However, we believe the most compelling evidence for disturbance by the UAS were the upflights observed immediately following takeoff on the first flight of the first two survey days, but not thereafter. This would seem to be consistent with a pattern of habituation [12] whereby the terns were initially alarmed by a novel stimulus but subsequently stopped reacting as the aircraft regularly reappeared over several days and repeatedly flew over the colony without adverse consequences. In any case, it can be confidently asserted that the UAS caused far less disturbance than the conventional ground census of the colony.

In summary, this study provides the most comprehensive evidence to date that small UAS can be effectively employed for fine-scale, low-disturbance population monitoring of colonial waterbirds. The technique could potentially be applied to several medium- to large-sized species that place their nests on the ground in relatively open habitats, such as other tern species, gannets (*Morus* spp.), cormorants (*Phalacrocorax* spp.) or gulls (Laridae spp.). Higher resolution cameras may also enable detection of smaller species such as Least auklets (*Aethia pusilla*) and small shorebirds. However, prospective users of the technology need carefully consider a number of criteria that could affect the ability to obtain accurate population estimates. Compared to the Common tern colony surveyed in our study, other colonies or species may vary in: (1) nest attendance rates; (2) nesting synchrony, which could further complicate assumptions about nest attendance; (3) the ability to identify pairs attending a single nest; (4) the ability to distinguish between loafing birds and those attending nests; (5) the ability to distinguish between species in multi-species colonies; (6) the visibility of birds in terms of their contrast with the substrate, as previously noted by Chabot and Bird [16]; and (7) their tolerance of overflights by UAS. We therefore recommend that calibration of aerial against ground counts be conducted to some extent on a species-by-species and site-by-site basis in order to assess and control for the above variables. A key advantage of small UAS is that repeated surveys required to accomplish this can be conducted at relatively little extra cost [17].

Use of UAS can additionally save time and effort in the field compared to conducting ground surveys: the total ground nest count carried out on Tern Islands took a dozen surveyors approximately four hours to complete, whereas the total time required for two operators to set up the UAS, execute a single flight over the colony and subsequently pack up did not exceed 90 minutes. However, the operation of small lightweight UAS is typically constrained by wind conditions and precipitation. For instance, most of our surveys of the coastal colony were restricted to the early morning when winds tended to be calmer. We were also fortunate that the South Kouchibouguac Dune (Fig 1) provided a conveniently located landing strip directly adjacent to Tern Islands. Many small fixed-wing UAS such as the AI-Multi require a generous runway for landings while others are equipped with a parachute, although this would have been hazardous over the narrow dune since the aircraft could have drifted in the wind following parachute deployment and potentially landed in the water. Small rotary-wing UAS can conveniently take off and land vertically in tight spaces, although they are typically limited to about half the flight endurance of fixed-wing models of equivalent size, and it remains to be determined whether birds are similarly tolerant of rotary-wing models.

As they continue to become more widespread and accessible, UAS are anticipated to play increasingly frequent and varied roles in wildlife and ecological science [15,32,33]. An important consideration for all prospective UAS users is airspace regulations, which can be restrictive or prohibitive in most developed countries. However, many governments are now in the process of updating regulations in order to better accommodate UAS, recognizing the inevitability of their proliferation [34,35]. In the present study we purchased and learned to operate the UAS, obtained authorization to fly it, and processed and analyzed the data ourselves, an accomplishment requiring a highly specialized skill set that took several years to develop. As an alternative, a commercial service sector is beginning to emerge offering on-demand UAS data collection and processing to customers who are interested in taking advantage of the technology but unable or unwilling to purchase and learn to operate their own systems [36]. We encourage waterbird researchers and managers to explore the potential benefits of using this burgeoning technology for surveying colonially breeding species.

## Supporting Information

S1 Photo Sample unmanned aerial photograph of a Common tern breeding colony. Imagery was acquired from 91 m in altitude by a Canon PowerShot S90 on-board an Aerial Insight AI-Multi small unmanned aircraft system (UAS) over Tern Islands, Kouchibouguac National Park, New Brunswick, 2012. The image has a ground footprint of approximately 113 m by 85 m and a resolution of ~3 cm/pixel.
(JPG)

S1 Video Launch and landing of the Aerial Insight AI-Multi electric unmanned aircraft. (M4V)
