# Supplementary figures and images for: Population Census of a Large Common Tern Colony with a Small Unmanned Aircraft
Source: PLoS One. 2015 Apr 15;10(4):e0122588. doi: 10.1371/journal.pone.0122588 (PMC4398491; doi:10.1371/journal.pone.0122588)

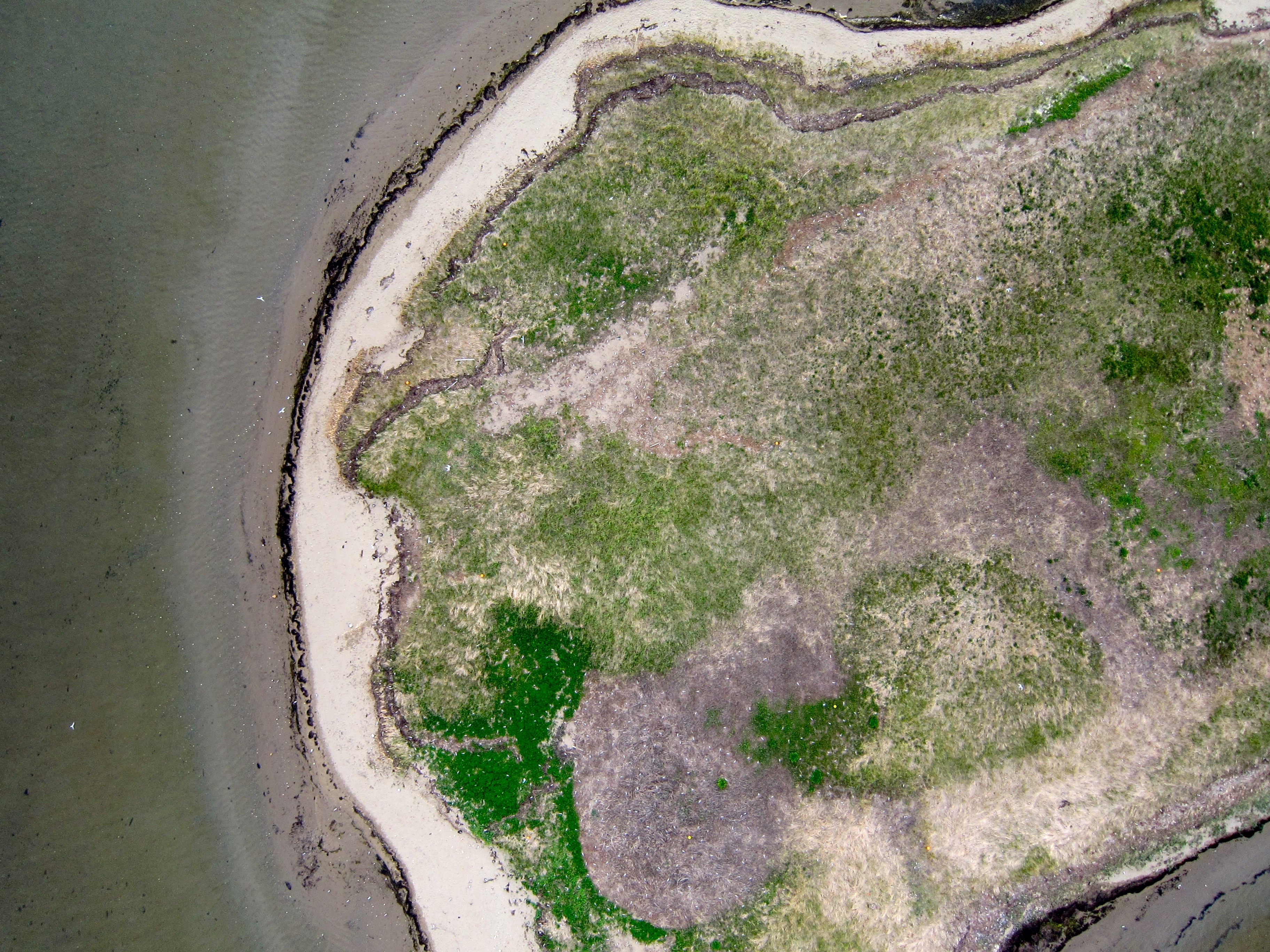

Supplement: S1 Photo — Imagery was acquired from 91 m in altitude by a Canon PowerShot S90 on-board an Aerial Insight AI-Multi small unmanned aircraft system (UAS) over Tern Islands, Kouchibouguac National Park, New Brunswick, 2012. The image has a ground footprint of approximately 113 m by 85 m and a resolution of ~3 cm/pixel. (JPG) [file pone.0122588.s001.jpg]
